# Realization of broadband negative refraction in visible range using vertically stacked hyperbolic metamaterials

**DOI:** 10.1038/s41598-019-50434-3

**Published:** 2019-10-01

**Authors:** Sanghun Bang, Sunae So, Junsuk Rho

**Affiliations:** 1 Department of Mechanical Engineering, Pohang University of Science and Technology (POSTECH), Pohang, 37673 Republic of Korea; 2 Department of Chemical Engineering, Pohang University of Science and Technology (POSTECH), Pohang, 37673 Republic of Korea; 3 National Institute of Nanomaterials Technology (NINT), Pohang, 37673 Republic of Korea

**Keywords:** Metamaterials, Nanophotonics and plasmonics

## Abstract

Negative refraction has generated much interest recently with its unprecedented optical phenomenon. However, a broadband negative refraction has been challenging because they mainly involve optical resonances. This paper reports the realization of broadband negative refraction in the visible spectrum by using vertically-stacked metal-dielectric multilayer structures. Such structure exploits the characteristics of the constituent metal and dielectric materials, and does not require resonance to achieve negative refraction. Broadband negative refraction (wavelength 270–1300 nm) is numerically demonstrated. Compared to conventional horizontally-stacked multilayer structures, the vertically-stacked multilayer structure has a broader range of working wavelength in the visible range, with higher transmittance. We also report a variety of material combinations with broad working wavelength. The broadband negative refraction metamaterial provides an effective way to manipulate light and may have applications in super-resolution imaging, and invisibility cloaks.

## Introduction

Metamaterials composed of nanostructures of artificial atoms have shown many extraordinary properties that cannot be found in natural materials^1,2^. Super-resolution imaging^3,4^, negative index materials (NIMs)^5–7^, invisibility cloak^8,9^, perfect absorbers^10–12^, artificial chirality^13,14^, and electromagnetically induced transparency^15^ are examples of metamaterial applications that have properties beyond the limits of natural materials. Hyperbolic metamaterials (HMMs) show distinctive characteristics such as negative refraction^16,17^, sub-wavelength imaging^18–20^, confining the electromagnetic field^21^, and thermal emission^22,23^.

In natural materials, permittivity *ε* and permeability *μ* cannot be negative simultaneously, but if a certain material has negative values of both *ε* and *μ*^24^, the refractive index *n* becomes negative. The conventional materials with positive refractive index *n* have a positive value of the angle of refraction *θ*_2_ according to Snell’s law:

1 $${n}_{1}\,\sin \,{\theta }_{1}={n}_{2}\,\sin \,{\theta }_{2}$$

In contrast, NIMs show a negative *θ*_2_; i.e., negative refraction. The first NIM was achieved using split-ring resonators (SRRs), which have both negative *ε* and negative *μ*^25^. The negative *ε* is obtained by conducting wires below the plasma frequency *ω*_P_, and negative *μ* occurs near the resonance frequency *ω*_SRR_ of SRRs. If *ω*_P_ > *ω*_SRR_, then *ε* and *μ* are simultaneously negative in a certain region. Multilayered fishnet structure^7^, chiral structure^26–28^, and asymmetry nanorings structure^29^ have been studied to achieve negative *n*. NIMs have provided the possibility of applications such as super-resolution imaging and invisibility cloaks.

Conventional metamaterials for NIMs^30,31^ have some drawbacks. The materials require optical resonance to obtain a negative *μ*; this requirement restricts the range of working wavelength Δλ_W_ to a narrow region near the resonance and causes high resistive loss. As a consequence, development of practical applications has been obstructed. However, electrostatic and magnetostatic fields can be decoupled in electrostatic limits, in which a system has smaller dimensions than the wavelength^6^. Therefore, under transverse magnetic (TM) polarized light, the electromagnetic behaviors in such system are only relevant to the permittivity. The HMMs consist of constituents with sub-wavelength dimensions, so negative refraction under TM polarized light can be achieved in HMMs by simply manipulating *ε* even if they do not have negative *n*.

So far, the negative refraction obtained from HMMs has mainly been shown with horizontally stacked metal-dielectric multilayer structures^7,32–34^ (horizontal HMMs). Such structure use an optical resonance to attain negative refraction; this requirement narrows Δλ_W_ and causes high resistive losses. However, realization of a broad Δλ_W_ and low-loss requires that resonance be avoided. Therefore, in this paper, we present vertically-stacked metal-dielectric multilayer structures (vertical HMMs) to achieve broadband negative refraction. This structure does not require any resonance to realize negative refraction and therefore it has a broad Δλ_W_ that includes the visible range without additional losses due to resonance. The dispersion relation of HMMs was derived using effective medium theory^35,36^ (EMT) and using this relation, the Δλ_W_ of negative refraction and transmittance of the vertical HMMs and horizontal HMMs are compared. We also suggest various vertical HMMs composed of different metal and dielectric materials. Plots of working wavelength and filling ratio of several metals with fixed dielectric show broadband Δλ_W_ in the vertical HMMs.

## Results

**Figure 1:**
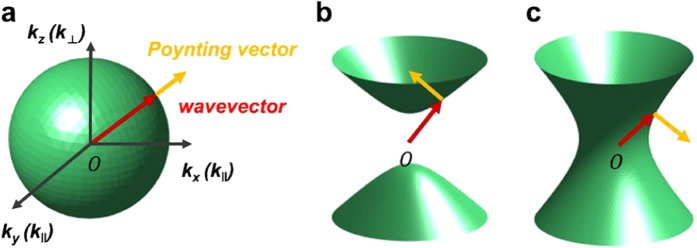
Diagram of the isofrequency surface of each material. (**a**) Isotropic materials, in which the permittivity is the same along all directions, have spherical isofrequency. (**b**) Isofrequency surface of a type-I HMM, which has positive permittivity along the layers (***ε***_***xx***_, ***ε***_***yy***_) and negative permittivity in the vertical direction (***ε***_***zz***_). (**c**) Isofrequency surface of a type-II HMM, which has negative permittivity along the layers (${{\boldsymbol{\varepsilon }}}_{{\boldsymbol{xx}}},\,{{\boldsymbol{\varepsilon }}}_{{\boldsymbol{yy}}}$) and positive permittivity in the vertical direction (***ε***_***zz***_). Red arrow: wavevector $\overrightarrow{{\boldsymbol{k}}}$; yellow arrow: Poynting vector $\overrightarrow{{\boldsymbol{S}}}$ that denotes energy flow. In isotropic materials, $\overrightarrow{{\boldsymbol{k}}}$ and $\overrightarrow{{\boldsymbol{S}}}$ point in the same direction; in type-I (**b**) and type-II (**c**) HMMs the vectors point in different directions.

Multilayer structures are uniaxial media, in which permittivity tensors consist of only diagonal components. Among the components, the permittivity along the layers is the same (${\varepsilon }_{xx}={\varepsilon }_{yy}$). Therefore, the dispersion relation for TM polarized light ($\overrightarrow{k}\cdot \overrightarrow{H}=0$) for multilayer structure is described as

2 $$\frac{{k}_{x}^{2}+{k}_{y}^{2}}{{\varepsilon }_{\perp }}+\frac{{k}_{z}^{2}}{{\varepsilon }_{\parallel }}={k}_{0}^{2}={(\frac{\omega }{c})}^{2},$$

where *k*_*x*_, *k*_*y*_, *k*_*z*_ are directional wavevectors in the medium, *k*_0_ is a wavevector in vacuum, *ω* is the frequency of the wave, *c* is the velocity of light in vacuum, ${\varepsilon }_{\parallel }$ is permittivity along the layers (${\varepsilon }_{xx}={\varepsilon }_{yy}$), and ${\varepsilon }_{\perp }$ is permittivity perpendicular to the layers (*ε*_*zz*_). An isotropic medium with the same permittivity in all direction has a spherical isofrequency surface, which shows the isotropic behavior of propagating waves (Fig. 1a). In contrast, in an anisotropic medium, ${\varepsilon }_{\parallel }$ and ${\varepsilon }_{\perp }$ are different, so the isofrequency surface of is not spherical^37^. In the multilayer structure, each directional permittivity can be replaced by an effective permittivity, which is calculated using effective medium theory^36^. This theory considers an anisotropic composite as a homogeneous medium with effective parameters.

If a multilayer structure consists of alternating metal and dielectric layers that have thickness much smaller than the wavelength, this structure can be regarded as a homogeneous medium. Effective permittivities differ according to the direction (Supplementary Note 1):

3 $${\varepsilon }_{\parallel }={\varepsilon }_{d}f+{\varepsilon }_{m}(1-f),$$

4 $${\varepsilon }_{\perp }=\frac{{\varepsilon }_{d}{\varepsilon }_{m}}{{\varepsilon }_{m}f+{\varepsilon }_{d}(1-f)},$$

where *f* is the filling ratio of the dielectric (*f* = 1 means that the medium is made entirely of a dielectric; *f* = 0 means that the medium is made entirely of a metal), *ε*_*d*_ is the permittivity of dielectric, and *ε*_*m*_ is the permittivity of the metal. With the effective permittivity, Eq. (2) becomes an elliptic or a hyperbolic equation. Eq. (2) describes a hyperbolic isofrequency surface if the signs of the ${\varepsilon }_{\parallel }$ and ${\varepsilon }_{\perp }$ are different, where multilayer structure shows a dielectric or a metallic behavior according to the direction. If ${\varepsilon }_{\perp }$ is the negative permittivity, the HMM is classified as type-I; if ${\varepsilon }_{\parallel }$ is negative, the HMM is classified as type-II (Fig. 1b,c). Therefore, HMMs are considered as extremely anisotropic materials that have hyperbolic isofrequency surfaces^38^.

Conventional HMMs are usually made by horizontally stacking metal and dielectric, and are of type-I HMMs. However, this structure uses resonance to realize negative refraction, and therefore has limitations such as narrow Δλ_W_. To overcome this limitation, we suggest a structure in which metal and dielectric are vertically stacked alternately. This structure has a negative parallel effective permittivity and a positive perpendicular effective permittivity, and is therefore classified as a type-II HMM. The cause of each negative permittivity should be understood. ${\varepsilon }_{\parallel }$ and ${\varepsilon }_{\perp }$ reach negative values by distinct mechanisms. The permittivity *ε*_*m*_ of metal can be described by the Drude model^39–41^ and the permittivity *ε*_*d*_ of the dielectric can be considered to be constant regardless of *ω*.

5 $${{\rm{\varepsilon }}}_{m}=1-\frac{{\omega }_{p}^{2}}{{\omega }^{2}+i\gamma \omega },$$

where *ω*_*p*_ is a plasma frequency and *γ* is damping ratio. The effective permittivity ${\varepsilon }_{\parallel }$ along the layers is the arithmetic mean of permittivities of constituents (Eq. 3); this is similar to the permittivity equation from the Drude model^42^. In contrast, the effective permittivity ${\varepsilon }_{\perp }$ perpendicular to the layers is the harmonic mean of permittivities of each component (Eq. 4); the form is similar to the permittivity equation from the Lorentz model.

6 $${\rm{\varepsilon }}=1+{\rm{\chi }}+\frac{{{\rm{Ne}}}^{2}}{{\varepsilon }_{0}{m}_{0}}\cdot \frac{1}{{\omega }_{0}^{2}-{\omega }^{2}-i\gamma \omega },$$

where *χ* is a susceptibility, N is the number of atoms per unit volume, *e* is the magnitude of the electric charge of the electron, *m*_0_ is the mass of the electron, and *ω*_0_ is resonance frequency. As a result, ${\varepsilon }_{\parallel }$ and ${\varepsilon }_{\perp }$ vary with *ω*. Analysis of the models reveals that the real value of ${\varepsilon }_{\parallel }$ does not require resonance to be negative if the wavelength of incident light is larger than the wavelength that corresponds to the effective *ω*_P_ of metal (${\rm{\lambda }} > {{\rm{\lambda }}}_{{\rm{ep}}}$), whereas the real value of ${\varepsilon }_{\perp }$ is negative when the wavelength is near Lorentz resonance (${\rm{\lambda }}\simeq {{\rm{\lambda }}}_{res}$). Therefore, horizontal HMMs that need negative ${\varepsilon }_{\perp }$ use resonance to realize negative refraction^43^ whereas vertical HMMs that need negative ${\varepsilon }_{\parallel }$ achieve negative refraction without using resonance.

**Figure 2:**
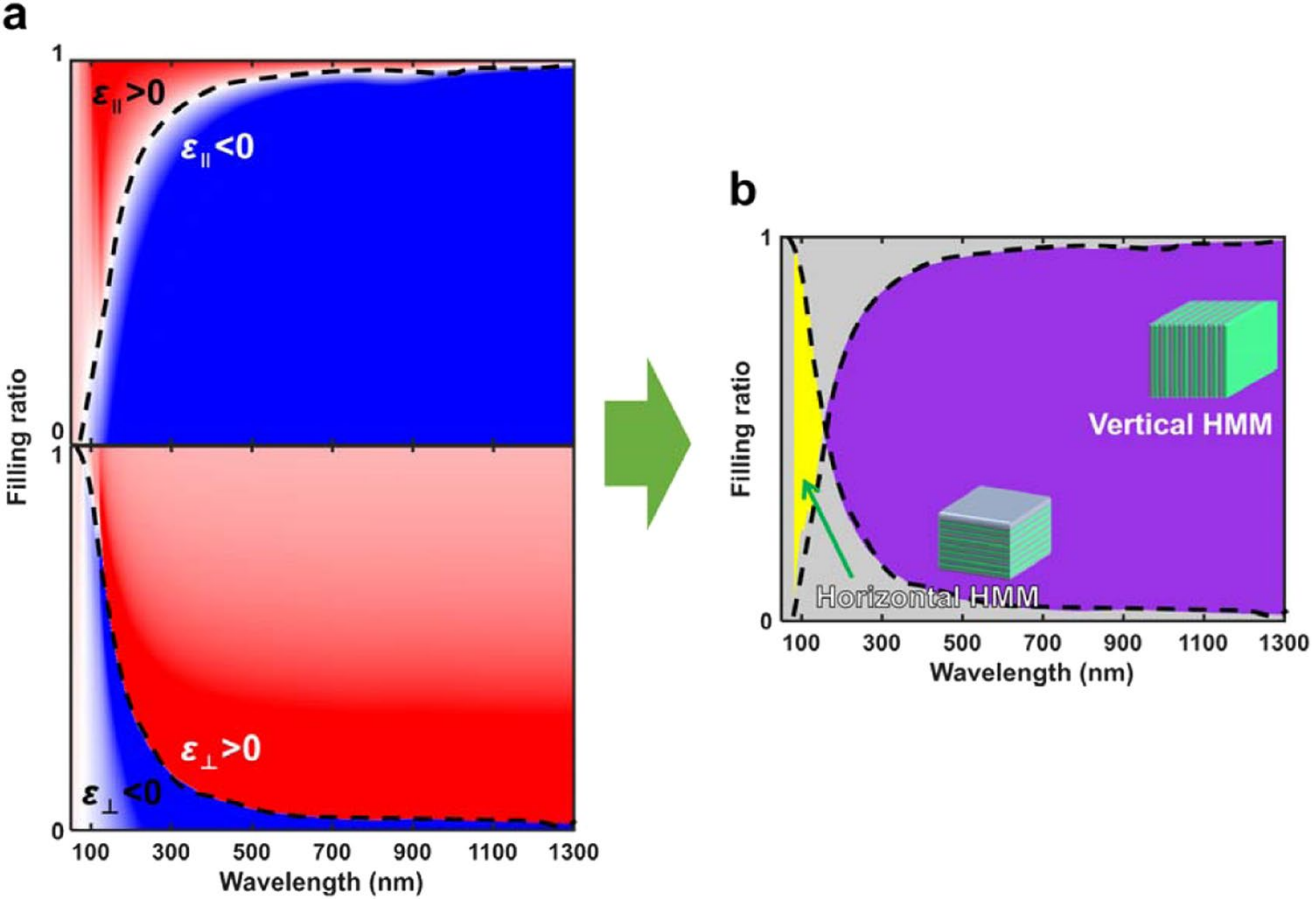
Diagrams of effective permittivity and the working wavelength Δλ_W_ of a vertical HHM composed of Al and SiO_2_. Effective permittivity (**a**, upper) ${{\rm{\varepsilon }}}_{\parallel }$ along the layer and (**b**, lower) ${{\rm{\varepsilon }}}_{\perp }$ perpendicular to the layer. Red regions: positive effective permittivity; blue regions: negative effective permittivity. (**b**) Effective permittivity (yellow) in type-I (horizontal) HMMs, which have narrow Δλ_W_; and (purple) in type-II (vertical) HMMs, which have a broad Δλ_W_ that includes the visible range.

The horizontal and vertical HMM composed of Al and SiO_2_ are used as an example to compare Δλ_W_ for negative refraction. The calculated effective permittivities (Fig. 2a) of Al and SiO_2_ multilayer structures have ranges of wavelength and filling ratio in which ${{\rm{\varepsilon }}}_{\parallel }$ is positive (red) and ${{\rm{\varepsilon }}}_{\perp }$ is negative (blue); this is Δλ_W_ for negative refraction in horizontal HMMs (Fig. 2b, yellow). These ranges also show an area in which ${{\rm{\varepsilon }}}_{\parallel }$ is negative (blue) and ${{\rm{\varepsilon }}}_{\perp }$ is positive (red); this is Δλ_W_ for negative refraction in vertical HMMs (Fig. 2b, purple). Compared to horizontal HMMs, the vertical HMMs have a broader Δλ_W_ that includes the visible range.

**Figure 3:**
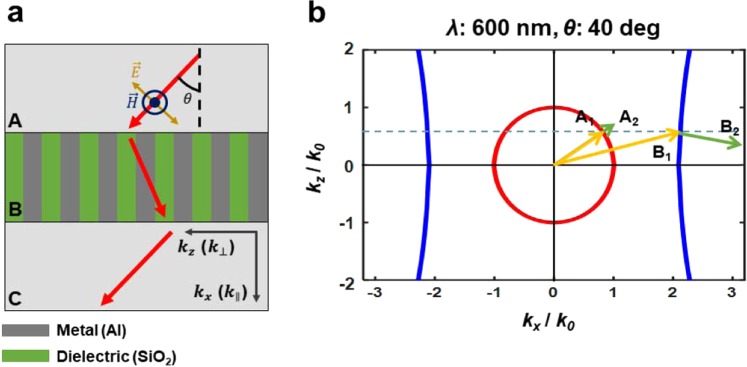
(**a**) Schematic of light propagation (red arrows) in vertical HMM. (**b**) 2D isofrequency surface for TM waves (blue curves) and isofrequency surface in the air (red circle) at 600 nm. Yellow arrows: wavevectors A_1_ in air and B_1_ in the HMM; green arrows: Poynting vector A_2_ in air and B_2_ in the HMM. When light is incident at a certain angle from air to the vertical HMM, the tangential component *k*_z_ of $\overrightarrow{{\boldsymbol{k}}}$ should be continuous at the boundary and the causality theorem must be satisfied, so only one solution is physically correct. B_1_ and B_2_ have different directions in *z* direction; this relationship means that vertical HMMs have negative refraction.

The negative refraction of an HMMs gives it a unique ability to manipulate light. In air, the wavevector $\overrightarrow{{\boldsymbol{k}}}$ and the Poynting vector $\overrightarrow{{\boldsymbol{S}}}$ of a TM wave are parallel, but when the TM wave meets the interface between the air and the HMM, $\overrightarrow{{\boldsymbol{k}}}$ and $\overrightarrow{{\boldsymbol{S}}}$ are not parallel. Consider a type-II HMM (i.e., ${\varepsilon }_{\parallel } < 0$ and ${\varepsilon }_{\perp } > 0$) and a wave that is propagating from air to HMM in the *x*-*z* plane; *i*.*e*., *k* is in the *x*-*z* plane (Fig. 3a). According to the continuity of tangential component (*k*_z,air_ = *k*_z,HMM_) and the causality theorem (${S}_{x} > 0$)^24,44^, only one solution exists (Fig. 3b, arrow B). The tangential component S_z_ of $\overrightarrow{{\boldsymbol{S}}}$ is in the opposite direction of that of $\overrightarrow{{\boldsymbol{k}}}$, so the HMM shows negative refraction. The exact direction of $\overrightarrow{{\boldsymbol{S}}}$ can be obtained using the Maxwell equations and some calculations (Supplementary Note 2).

Loss and transmittance of HMMs are related to resonance, which make a difference in performance between two types of HMMs. Horizontal HMMs that use resonance suffer high losses from it, so they have a narrow Δλ_W_ in which refraction is negative. In contrast, vertical HMMs do not use resonance, so they have wide Δλ_W_ and show no losses due to resonance^2^. Moreover, transmission is higher in vertical HMMs than in horizontal HMMs. Even though the transmission varies depending on wavelength, the combinations of metal and dielectric and the filling ratio, it can be represented roughly by the imaginary part of effective permittivity. As the imaginary part increases, the losses also increase^11^:

7 $${\rm{Total}}\,{\rm{power}}\,{\rm{dissipation}}\,{\rm{density}}({\rm{Loss}})=\frac{1}{2}{\varepsilon }_{0}{\varepsilon ^{\prime\prime} }_{r}\omega {|E|}^{2},$$

where *ε*_0_ is the permittivity of the vacuum, ω is the angular frequency, ${\varepsilon ^{\prime\prime} }_{r}$ is the imaginary part of the relative permittivity of the medium, and E is the electric field. Near the resonance frequency, the permittivity of material has a high imaginary part that induces a high absorption and thereby high resistive loss. On the other hand, the loss of the vertical HMMs is rarely affected by resonance because the vertical HMMs do not use the resonance for negative refraction; the loss of vertical HMMs is only related to constituent materials.

The horizontal HMM has a large imaginary part which leads to high losses because ${\varepsilon }_{\perp }$ of this HMM uses resonance to attain a negative real value. Therefore, high losses due to the resonance are inevitable in horizontal HMMs. However, each permittivity of vertical HMM has a small imaginary part because this HMM is not related to any resonance. In Ag-SiO_2_ multilayer structures, ${\varepsilon }_{\perp }$ in the horizontal HMM has a large imaginary part (${\varepsilon }_{\perp }$: −15.546 + 24.227i at 356 nm), whereas ${\varepsilon }_{\parallel }\,$in the vertical HMM has a small value (${\varepsilon }_{\parallel }$: −6.589 + 0.219i at 600 nm). Therefore, the vertical HMM shows a broader Δλ_W_ for negative refraction and higher transmittance than the horizontal type or conventional NIMs that use resonance.

**Figure 4:**
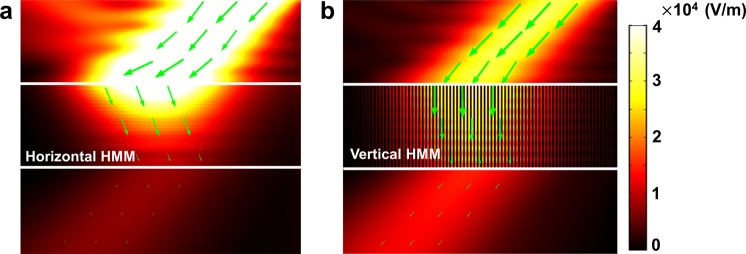
Intensity profile in horizontal and vertical HMM composed of Al and SiO_2_. Surface color represents normalized electric field [V/m]; green arrows: Poynting vector that indicates the direction of energy flow. The height of each HMM was ~2.5 times the wavelength used in the simulations. (**a**) Horizontal HMM. Light with wavelength 135 nm is incident with an angle of 40° to the horizontal HMM; the filling ratio is 0.5, each layer is 6.75 nm thick, and the total height is 330 nm. (**b**) Vertical HMM. Light with wavelength 600 nm is incident with an angle of 40°; the filling ratio is 0.5, each layer is 30 nm thick, and the total height is 1,500 nm.

We also compared two types of HMMs in terms of transmittance and intensity profiles (Fig. 4). Al and SiO_2_ multilayered structure with 0.5 filling ratio is used in each simulation because it has a wide Δλ_W_ that includes the whole visible range. According to the Δλ_W_, the horizontal HMM was simulated at 135 nm and the vertical structure was simulated at 600 nm in which it shows negative refraction. The layer thickness was 6.75 nm in the horizontal HMM and 30 nm in the vertical HMM. The total height of each structure was determined by how many times the wave proceeds, so thickness of ~2.5λ was used for each simulation. The light is incident at an angle of 40°. In the simulation results, the transmittance is 0.01 in the horizontal HMM and 0.18 in the vertical HMM. The results clearly show that the vertical HMM has a higher transmittance than the horizontal one. Although the absolute value of transmittance by vertical HMMs is not high, it is enough to allow utilization of negative refraction.

**Figure 5:**
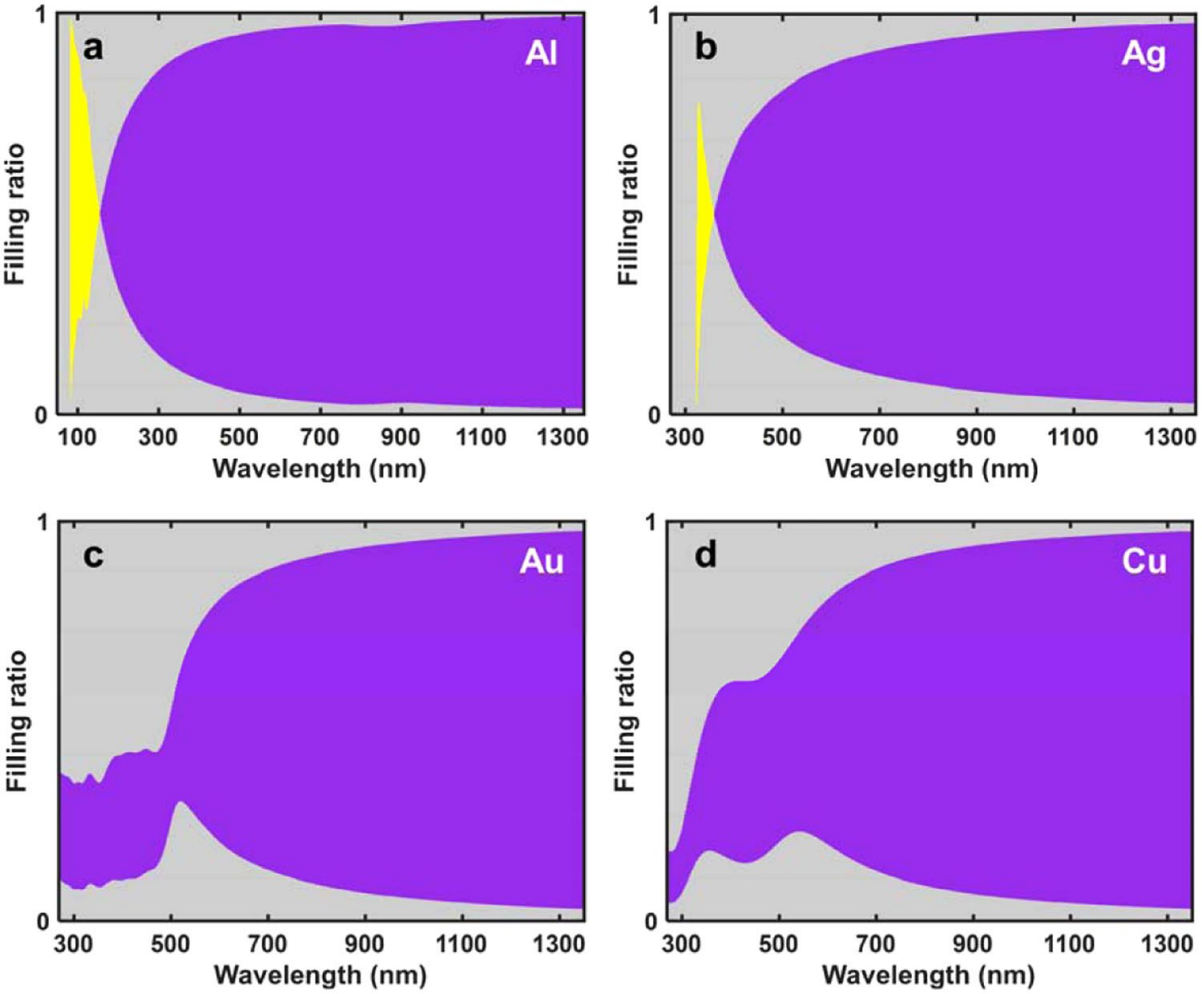
Effects of filling ratio *f* and wavelength. Various metals are used with same dielectric SiO_2_. When a medium is made entirely of dielectric, *f* = 1, when the medium is made entirely of metal, *f* = 0. Yellow region: Δλ_W_ for negative refraction in type-I HMMs; purple regions: Δλ_W_ for negative refraction in type-II HMMs. When *f* = 0.5, (**a**) Al is type-I at 85 ≤ λ ≤ 155 nm and type-II at λ > 155 nm. (**b**) Ag was type-I at 330 ≤ λ ≤ 360 nm and type-II at λ > 360 nm, (**c**) Au only type-II at λ> 500 nm, and (**d**) Cu is only type-II at λ > 350 nm. Type-II region of HMMs of all combination shows a larger Δλ_W_ that includes the visible range.

Various material combinations of the vertical HMMs with Ag, Al, Au, and Cu as a metal and SiO_2_ as a dielectric were also analyzed (Fig. 5). Each combination is plotted by wavelength and filling ratio to compare the working wavelength, which is denoted by yellow and purple regions. The yellow and purple regions represent the working range for negative refraction in the type-I HMMs and the type-II HMMs, respectively. It is noteworthy that aAll combinations of the materials has much broader Δλ_W_ in the vertical HMMs than in horizontal HMMs, and that some combinations can realize negative refraction only in vertical HMMs. (transmittances in Supplementary Note 3). Therefore, various vertical HMMs which use type-II region have a potential to realize negative refraction over the visible wavelength range.

This paper has demonstrated a broadband negative refraction that was realized by vertically stacked metal-dielectric multilayer structures. These vertical HMMs do not use resonance to realize negative refraction, so they do not suffer from losses due to this resonance. Therefore, vertical HMMs have higher transmittance and Δλ_W_ that covers the entire visible range and extends into the mid-infrared without additional losses. We also provide several material combinations for broadband negative refraction that includes the visible range. Because of these advantages, vertical HMMs can manipulate light by exploiting negative refraction so they can be widely used in light-controlling devices such as invisibility cloaks and super-resolution imaging.

## Method

We used COMSOL Multiphysics 5.3 for numerical simulation and RCWA (in-house code) to calculate transmittance.

## Supplementary information

Supplementary Information
