# Supplementary material for: Realization of broadband negative refraction in visible range using vertically stacked hyperbolic metamaterials
Source: Sci Rep. 2019 Oct 1;9:14093. doi: 10.1038/s41598-019-50434-3 (PMC6773722; doi:10.1038/s41598-019-50434-3)
Supplement: Supplementary file 1 — Supplementary Information [file 41598_2019_50434_MOESM1_ESM.docx]

**Supplementary Information**

**Realization of broadband negative refraction in visible range using vertically stacked hyperbolic metamaterials**

Sanghun Bang^1,†^, Sunae So^1,†^, and Junsuk Rho^1,2,3,*^

^1^Department of Mechanical Engineering, Pohang University of Science and Technology (POSTECH), Pohang 37673, Republic of Korea

^2^Department of Chemical Engineering, Pohang University of Science and Technology (POSTECH), Pohang 37673, Republic of Korea

^3^National Institute of Nanomaterials Technology (NINT), Pohang 37673, Republic of Korea

^*^correspondence to [jsrho@postech.ac.kr](mailto:jsrho@postech.ac.kr)

^†^These authors contributed equally to this work.

# Supplementary Note 1. Effective medium theory: Maxwell-Garnett mixing formula

The Lorentz formula^S1^ for the permittivity of a nonpolar molecular gas is

$\varepsilon=1+\frac{4\pi(\alpha/\nu)}{(4\pi/3)(\alpha/\nu)}$, (S.1)

where $\alpha$ is the static polarizability, $\nu$ is the specific volume per molecule [V/N]. The Lorentz formula can be extended to the Maxwell Garnett mixing formula that provides a homogeneous permittivity *ε_MG_* of the complex composite medium. Suppose that small identical spherical inclusions with radius *a* and a relative permittivity $\varepsilon_{i}$ are embedded in a host medium $\varepsilon_{h}$ with a volume density $f$. The static polarizability^2^ for one dielectric sphere can be obtained by solving Laplace equation with proper boundary conditions at the spherical surface and at infinity,

$\alpha=a^{3}\frac{\varepsilon_{i}-1}{\varepsilon_{i}+2}$, (S.2)

and the volume density of inclusions is expressed

$f=\frac{4\pi}{3}\cdot\frac{a^{3}}{\nu}$. (S.3)

With the static polarizability (S.2) and the volume fraction (S.3), $\alpha/\nu$ is obtained.

$\frac{\alpha}{\nu}=\frac{3}{4\pi}f\cdot\frac{\varepsilon_{i}-1}{\varepsilon_{i}+2}$ (S.4)

Using equation (S.4), Maxwell-Garnett mixing formula can be obtained by putting it into equation (S.1).

$\varepsilon_{MG}=\varepsilon_{h}\frac{1+2f\frac{\varepsilon_{i}-\varepsilon_{h}}{\varepsilon_{i}+2\varepsilon_{h}}}{1-f\frac{\varepsilon_{i}-2\varepsilon_{h}}{\varepsilon_{i}+2\varepsilon_{h}}}=\varepsilon_{h}\frac{\varepsilon_{h}+\frac{1+2f}{3}(\varepsilon_{i}-\varepsilon_{h})}{\varepsilon_{h}+\frac{1-f}{3}(\varepsilon_{i}-\varepsilon_{h})}$, (S.5)

or

$\frac{\varepsilon_{MG}-\varepsilon_{h}}{\varepsilon_{MG}+2\varepsilon_{h}}=f\frac{\varepsilon_{i}-\varepsilon_{h}}{\varepsilon_{i}+2\varepsilon_{h}}$, (S.6)

$\varepsilon_{MG}$ is replaced by $\varepsilon_{MG}/\varepsilon_{h}$ and $\varepsilon_{i}$ is replaced by $\varepsilon_{i}/\varepsilon_{h}$ for the generalization of the host medium^S3^.

The formula (S.6) applies only to isotropic composites, so a generalized formula is needed for anisotropic composites. In an anisotropic composite, values of the effective permittivity differ depending on directions, so it becomes a tensor $\hat{\varepsilon}_{MG}$. Although effective permittivity is expressed by a tensor, it can be considered simply as a diagonal tensor due to a symmetry property. To represent anisotropic composites, the inclusions are assumed to be identical, uniformly distributed and similarly oriented ellipsoids without interaction. From the Laplace equation, the static polarizability of the inclusions becomes a tensor $\hat{\alpha}$, and principal components are represented as

$\alpha_{p}=\frac{a_{x}a_{y}a_{z}}{3}\frac{\varepsilon_{h}(\varepsilon_{i}-\varepsilon_{h})}{\varepsilon_{h}+\nu_{p}(\varepsilon_{i}-\varepsilon_{h})} \left( p=x,y,z \right)$, (S.7)

where $a_{x}, a_{y}, a_{z}$ are semi-axes that are parallel to the principal X, Y, Z axes respectively, $\nu_{p}$ are ellipsoid depolarization factors ($0<\nu_{p}<1$, $\nu_{x}+\nu_{y}+\nu_{z}=1$) that explain the dependence of the internal field and the permittivity of the ellipsoid according to the external electric field^S4^. The host medium is a large ellipsoid with semi-axes $R_{x}, R_{y}, R_{z}$ which are larger than $a_{x}, a_{y}, a_{z}$, and is filled uniformly with the inclusions. The large ellipsoid is considered to have an effective permittivity, so its polarizability become total polarizability such that

$\hat{\alpha}_{tot}=N\hat{\alpha}$, (S.8)

$\frac{R_{x}R_{y}R_{z}}{3}\frac{\varepsilon_{h}(\varepsilon_{MG}-\varepsilon_{h})}{\varepsilon_{h}+\nu_{p}(\varepsilon_{MG}-\varepsilon_{h})}=N\frac{a_{x}a_{y}a_{z}}{3}\frac{\varepsilon_{h}(\varepsilon_{i}-\varepsilon_{h})}{\varepsilon_{h}+\nu_{p}(\varepsilon_{i}-\varepsilon_{h})}$. (S.9)

With $fR_{x}R_{y}R_{z}=Na_{x}a_{y}a_{z}$, equation (S.9) can be arranged to

$\frac{\varepsilon_{MG}-\varepsilon_{h}}{\varepsilon_{h}+\nu_{p}(\varepsilon_{MG}-\varepsilon_{h})}=\frac{\varepsilon_{i}-\varepsilon_{h}}{\varepsilon_{h}+\nu_{p}(\varepsilon_{i}-\varepsilon_{h})}$. (S.10)

Rewriting this equation with respect to $\varepsilon_{MG}$ yields:^S1^

$\left( \varepsilon_{MG} \right)_{p}=\varepsilon_{h}\frac{\varepsilon_{h}+\left[ \nu_{p}\left( 1-f \right)+f \right](\varepsilon_{i}-\varepsilon_{h})}{\varepsilon_{h}+\nu_{p}\left( 1-f \right)(\varepsilon_{i}-\varepsilon_{h})}$, (S.11)

where *p* $\in$ {*x*,*y*,*z*} is a principal component of the effective permittivity tensor. If $\nu_{p}=1/3$, this equation becomes equal to the Maxwell Garnett mixing formula of isotropic composites.

The multilayer structure is satisfied in the extreme case where $\nu_{x}= \nu_{y}=0$ and $\nu_{z}=1$. In this case, the permittivity in the *x* and *y* directions along the layers is represented by

$\left( \varepsilon_{MG} \right)_{p}=f\varepsilon_{i}+\left( 1-f \right)\varepsilon_{h} when \nu_{p}=0,$ (S.12)

whereas a permittivity in the *z* direction perpendicular to the layers is

$\left( \varepsilon_{MG} \right)_{p}=\frac{\varepsilon_{i}\varepsilon_{h}}{f\varepsilon_{h}+\left( 1-f \right)\varepsilon_{i}} when \nu_{p}=1$. (S.13)

# Supplementary Note 2. Negative refraction in hyperbolic metamaterials

Negative refraction can be explained by the Maxwell equations when an EM wave propagates in a uniaxial material in which the electric permittivity tensor $\varepsilon$ consists of only diagonal terms i.e., $\varepsilon_{0}\left( \begin{matrix} \varepsilon_{xx} & 0 & 0 \\ 0 & \varepsilon_{yy} & 0 \\ 0 & 0 & \varepsilon_{zz} \end{matrix} \right)$. Then

$\nabla\times\vec{H}=-\varepsilon\frac{\partial\vec{E}}{\partial t}$. (S.14)

In the *x*-*y* plane, the electric field $\vec{E}$ and magnetic field $\vec{H}$ of a plane wave can be expressed as

$\vec{E}=\vec{E}_{0}e^{i(k_{x}x+k_{z}z-wt)}$, (S.15)

$\vec{H}=\vec{H}_{0}e^{i(k_{x}x+k_{z}z-wt)}$. (S.16)

This wave is TM so the H-field has only a y-component, and with Maxwell equation (S.14), $H_{0}$ is used to represent the E-field. Finally, the E-field and H-field are written as

$\vec{E}=\frac{H_{0}}{\omega\varepsilon_{0}}\left( \frac{k_{z}}{\varepsilon_{xx}}\hat{i}-\frac{k_{x}}{\varepsilon_{zz}}\hat{k} \right)e^{i\left( k_{x}x+k_{z}z-wt \right)}$, (S.17)

$\vec{H}=H_{0}\hat{j}e^{i(k_{x}x+k_{z}z-wt)}$. (S.18)

The Poynting vector represents the energy flows in the direction of wave propagation, and the time-averaged Poynting vector is calculated as

$<\vec{S}>=<\vec{E}\times\vec{H}>_{T}=\frac{1}{2}(\vec{E}\times\vec{H})$, (S.19)

Putting equations (S.17) and (S.18) into equation (S.19), yields normal S_x_ and tangential S_z_ components of Poynting vector are represented using wavevector *k* and permittivity $\varepsilon$^S5^.

$\left( S_{x},S_{z} \right)=\left( \frac{H_{0}^{2}}{2\omega\varepsilon_{0}}\frac{k_{x}}{\varepsilon_{z}},\frac{H_{0}^{2}}{2\omega\varepsilon_{0}}\frac{k_{z}}{\varepsilon_{x}} \right)$. (S.20)

Looking at the right term of S_x,_ both *k*_x_ and $\varepsilon_{z}$ are positive in HMM, so S_x_ is also positive. This result shows that the causality theorem is satisfied in this situation. In the tangential component case, S_z_ is negative because *k*_z_ is positive but $\varepsilon_{x}$ is negative in HMM. The negative value of S_z_ means the parallel direction of energy propagation is opposite to that of the incident wave. This result proves that negative refraction is possible by using HMM.

The accurate direction of the Poynting vector in HMMs is obtained by checking that the Poynting vector and the gradient of the hyperbolic isofrequency are vertical. The gradient of the hyperbolic isofrequency surface that corresponds to the wavevector $\left( k_{x},k_{z} \right)$ is obtained by differentiating equation (2) in the main text with respect to $k_{x}$ to yield

$\frac{dk_{z}}{dk_{x}}=\left( 1,-\frac{\varepsilon_{x}k_{x}}{\varepsilon_{z}k_{z}} \right)$. (S.21)

The inner product of the Poynting vector and the gradient of the surface is zero, which means that these two vectors are perpendicular to each other. Even though Poynting vector has two possible directions, only one is physically reasonable according to causality theorem. Therefore, the Poynting vector is normal to the gradient that corresponds to the wavevector in the hyperbolic isofrequency surface.

# Supplementary Note 3. Transmittance of material combinations

Transmittances of various vertical HMMs composed of different metal and dielectric combination with *f* = 0.5 were simulated in the visible range(Figure S1). Al, Ag, Au, and Cu were used as metals, and Al_2_O_3_, SiO_2_, HfO_2_, ZrO_2_, and TiO_2_ were used as the dielectric. The TM wave is incident at an angle of 40°. Each layer is 30 nm thick and total height is 1500 nm. RCWA (in-house code) is used for these simulations.


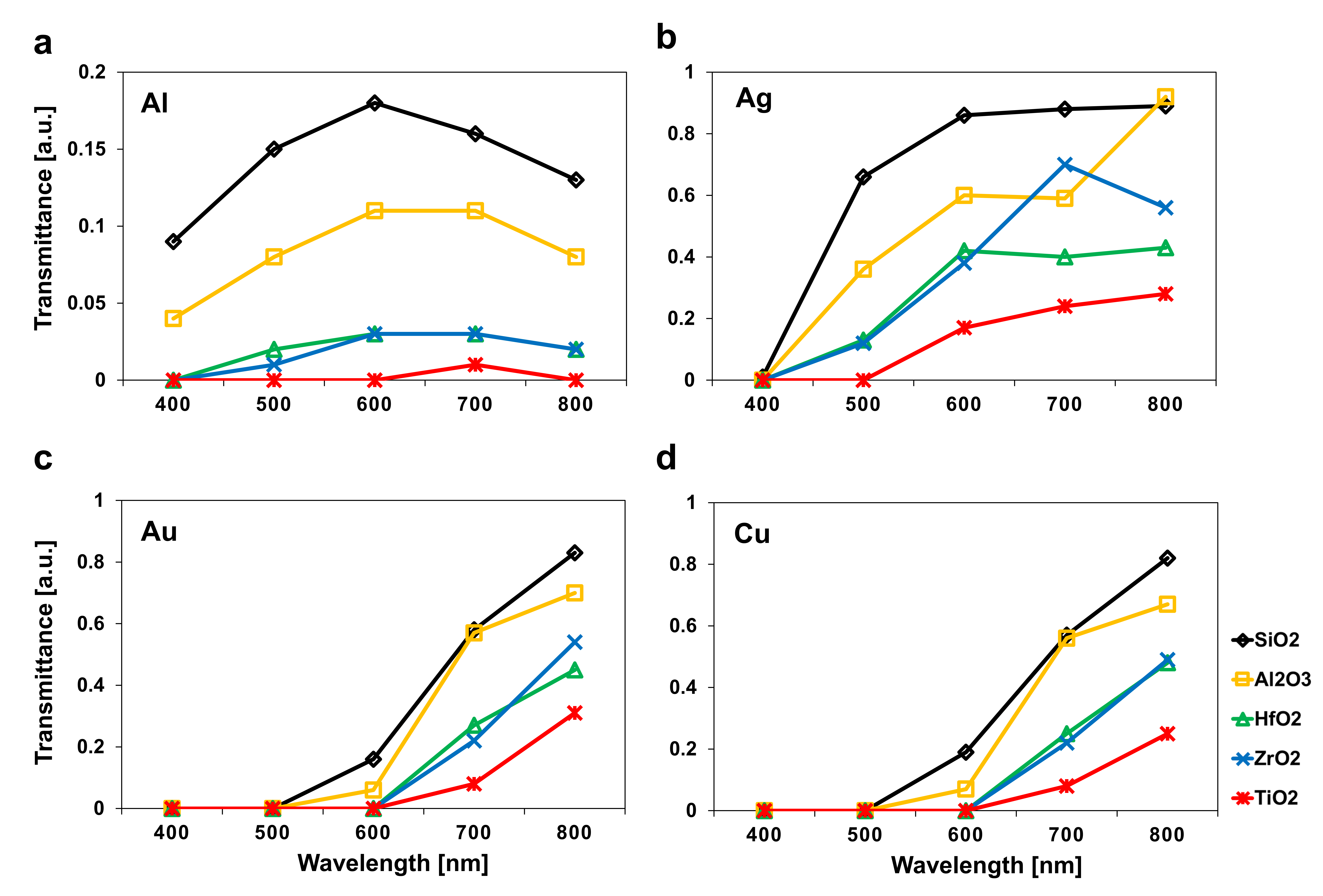


Figure S1. Transmittance of various metal and dielectric cases with 0.5 filling ratio

# Reference

S1 Markel, V. A. Introduction to the Maxwell Garnett approximation: tutorial. *JOSA A* **33**, 1244-1256, (2016).

S2 Ruppin, R. Evaluation of extended Maxwell-Garnett theories. *Opt. Commun.* **182**, 273-279, (2000).

S3 Niklasson, G. A., Granqvist, C. G. & Hunderi, O. Effective medium models for the optical properties of inhomogeneous materials. *Appl. Opt.* **20**, 26-30, (1981).

S4 Sihvola, A. Dielectric Polarization and Particle Shape Effects. *J. Nanomaterials* **2007**, 5-5, (2007).

S5 Liu, Y., Bartal, G. & Zhang, X. All-angle negative refraction and imaging in a bulk medium made of metallic nanowires in the visible region. *Opt. Express* **16**, 15439-15448, (2008).
